# Primary sacral non-Hodgkin’s lymphoma: report of a case and systematic review of literature

**DOI:** 10.1186/s12957-021-02153-1

**Published:** 2021-02-24

**Authors:** Satya Vijay Chigurupati, Mridula Shukla, Manoj Pandey

**Affiliations:** 1 Department of Surgical Oncology, Institute of Medical Science, Banaras Hindu University, Varanasi, 221005 India; 2 Dr. Lal Path Lab, Varanasi, India

**Keywords:** Primary bone lymphoma, Non-Hodgkin's lymphoma, Sacrum, Diffuse large B cell lymphoma

## Abstract

**Introduction:**

Isolated primary sacral diffuse large B cell non-Hodgkin’s lymphoma is a very rare entity, and only 11 cases have been reported previously.

**Case presentation:**

A 36-year-old man was referred with low backache and radiculopathy pain with a clinico-radiological and cytological diagnosis of sacral metastasis. Histopathological examination and immunohistochemistry of image-guided tissue core biopsy from the sacral mass confirmed it as high-grade diffuse large B cell lymphoma (DLBCL). With normal blood counts and bone marrow, and no lesions elsewhere on imaging, he was staged IAE and received 6 cycles of R-CHOP (rituximab, cyclophosphamide, doxorubicin, vincristine, and prednisolone) regimen chemotherapy followed by radiotherapy. The patient has completed a 3-year follow-up and is doing well with yearly imaging showing no evidence of active disease or recurrence.

**Conclusions:**

The case shows the importance of an image-guided core biopsy and immunohistochemistry over a fine needle aspiration cytology in select cases as it can alter the treatment and outcome in patients. Because of rarity, the treatment and prognosis in primary sacral NHL is not still very clear as it is treated as per the guidelines of treatment of bone lymphoma.

## Introduction

Primary bone lymphoma is a rare entity constituting less than 2% of all lymphomas in the adult population; it usually involves long bones of extremities [1, 2]. The sacrum is a very rare site for primary diffuse large B cell non-Hodgkin’s lymphoma (DLBCL), and a systematic search on PubMed showed only 11 reported cases [3–13]. For tumors to be considered as primary lymphoma of the bone, “it has to be a single skeletal site with or without regional lymph nodes or multiple bones involved with no visceral or lymph node involvement” [14]. We report a case of primary sacral lymphoma in a 36-year-old male who was referred with complaints of low backache and radiculopathy pain.

## Case report

**Fig. 1:**
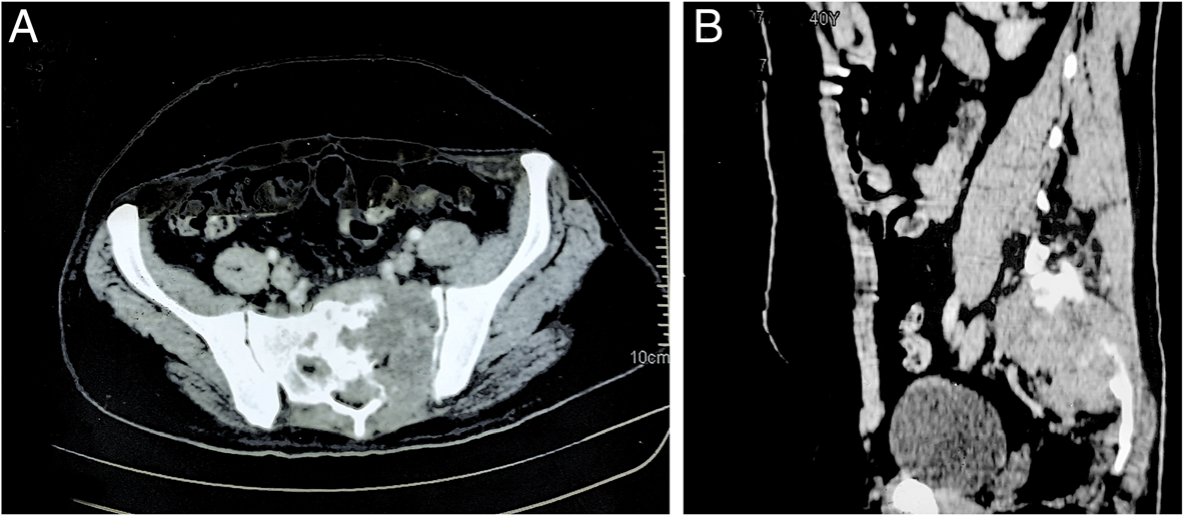
Pre-treatment CECT showing mass lesion in presacral region with underlying destruction of the sacrum in **a** sagittal and **b** axial views

A 36-year-old man was referred to us with a 30-day history of imbalance while walking, lower backache, tingling, and numbness in the left gluteal region radiating to the left lower limb. He was evaluated outside with a contrast-enhanced computed (CECT) scan suggesting a mass lesion in the presacral region with destruction and involvement of the underlying bone (Fig. 1). He also had an image-guided fine-needle aspiration from lesion suggestive of metastatic deposit.

**Fig. 2:**
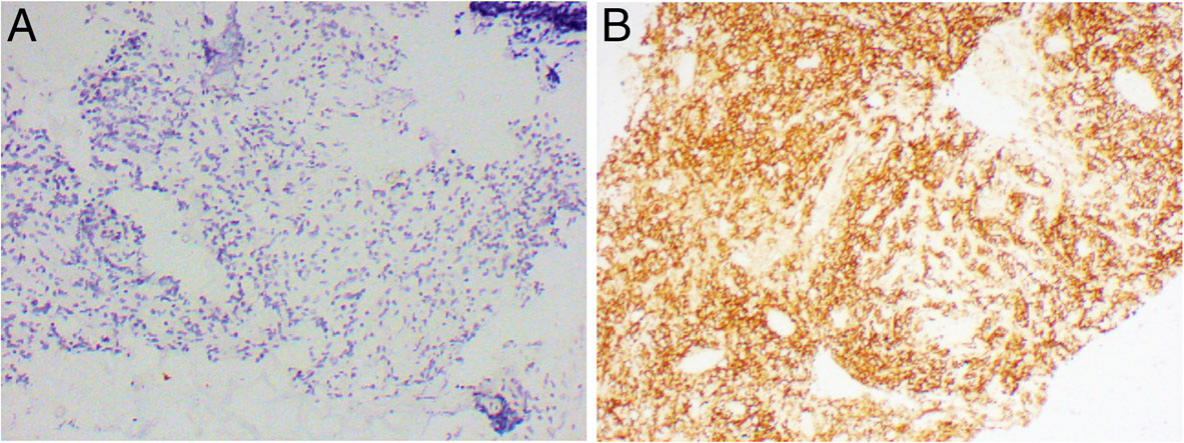
Photomicrograph showing **a** malignant round cells on hematoxylin and eosin staining (x10) and **b** cell immunostaining for CD20 (x10)

On clinical evaluation, the patient had an antalgic gait with weight-bearing on the right lower limb. Neurological examination showed normal higher mental functions and cranial nerves. Muscle power was 3/5 at left hip, 4/5 at the left knee, and ankle joints. Power in the right lower limb joints was normal. No sensory abnormalities were identified. The straight leg-raising test on the left side was restricted to 40° and was associated with sharp pain. Superficial and deep reflexes were normal in both limbs. The patient had neither bowel and urinary complaints nor history of fever, night sweats, or weight loss. Per abdomen, per rectal, and supraclavicular fossa examination revealed no abnormality. No clinically palpable nodes on systemic examination were found. With a tentative diagnosis of bone secondaries from an unknown primary, he was planned for image-guided core needle biopsy from the sacral mass that was carried out under full aseptic precautions. The histopathology (HPE) was suggestive of malignant round cell tumors. On immunohistochemistry (IHC), cells were positive for CD20 (score 4+), CD10 (score 1+), and ki67 (score 4+). The histological diagnosis was high-grade B cell non-Hodgkin lymphoma (NHL) compatible with diffuse large B cell lymphoma (Fig. 2). Hematology, biochemistry, and bone marrow study were normal. 18F-fluro-2-deoxy-d-glucose-positron emission tomography (FDG PET) scan showed lytic lesion in the sacrum with no other areas of FDG uptake.

**Fig. 3:**
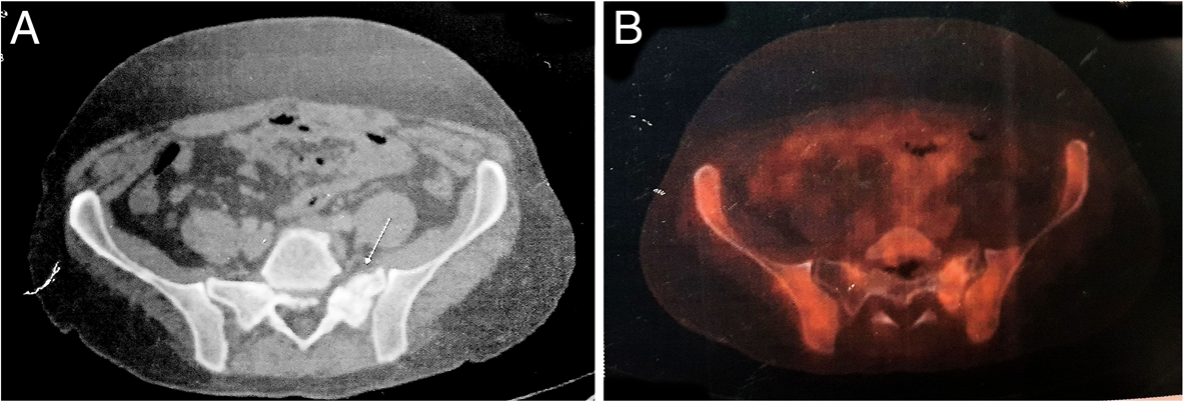
FDG-PET/CT in 2017 after 6 cycles of R-CHOP regimen. **a** Sclerotic lesion in left ala of the sacrum. **b** Mild FDG uptake in the left ala of sacrum (SUV of 3.22)

The patient was staged as IAE and started on rituximab, cyclophosphamide, doxorubicin, vincristine, and prednisolone (R-CHOP regimen). After completion of 3 weekly, 6 cycles, the patient was relieved of his pain and numbness and regained 5/5 power in the left lower limb muscles on clinical examination. Reevaluation by FDG-PET was done which showed a mild FDG avid sclerotic lesion of 1.3 × 1.2 cm in the left ala of the sacrum with standardized uptake value (SUV) of 3.22 suggesting a mild active residual disease (Fig. 3). He was started on maintenance rituximab and external photon radiotherapy to the sacrum (45Gy/25#) after multidisciplinary discussion.

**Fig. 4:**
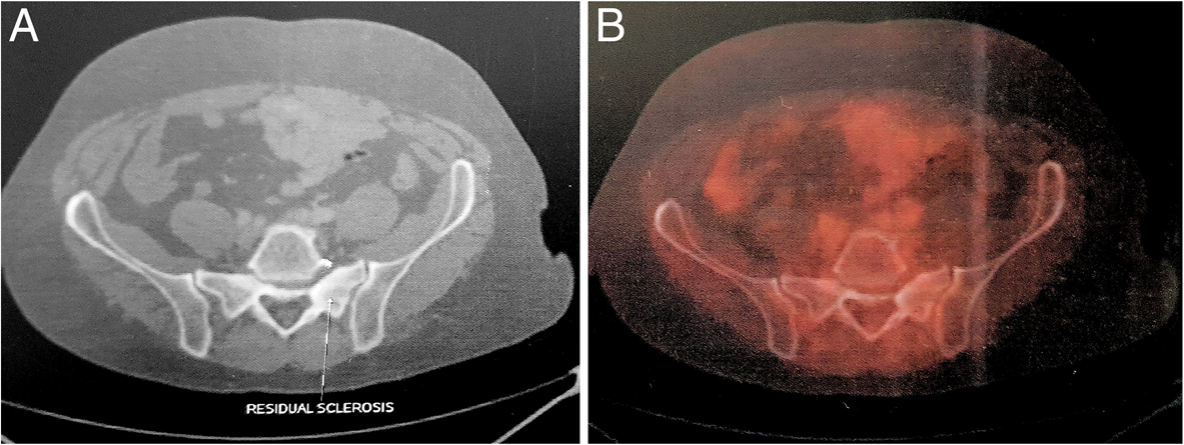
FDG-PET in 2017 after 6 cycles of R-CHOP regimen, external beam radiotherapy, and two cycles of maintenance rituximab. **a** Sclerosis at left ala of sacrum **b** with no metabolic uptake

**Fig. 5:**
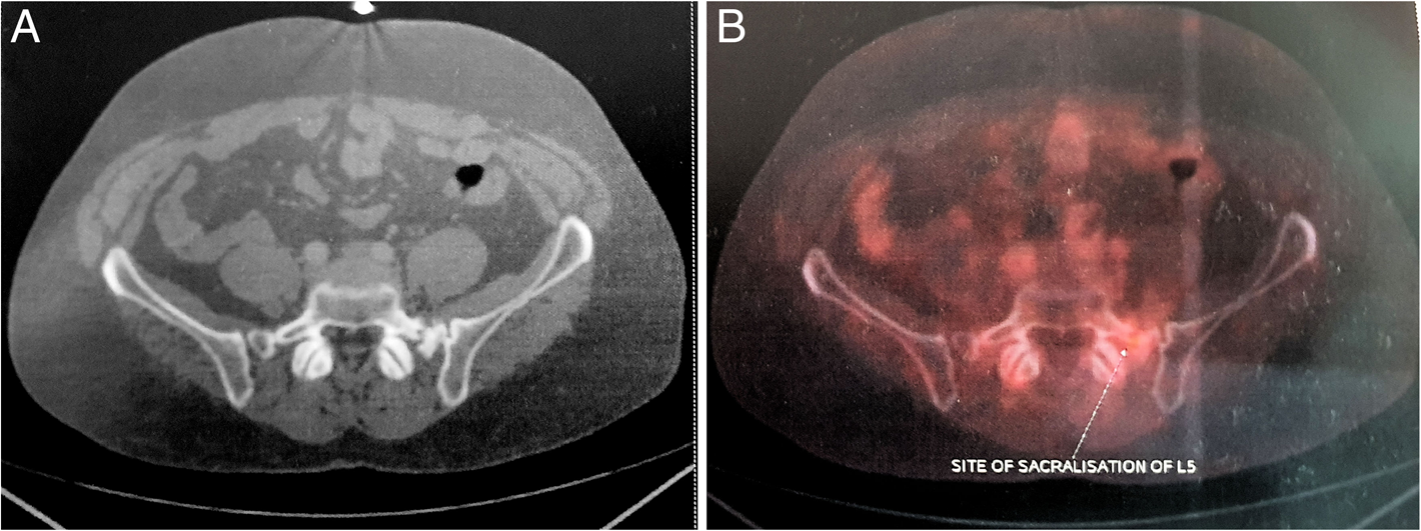
Follow-up FDG-PET/CT in 2018. **a** Sclerotic lesion in the left sacral ala. **b** Uptake noted at the site of sacralization (SUV 4)

**Fig. 6:**
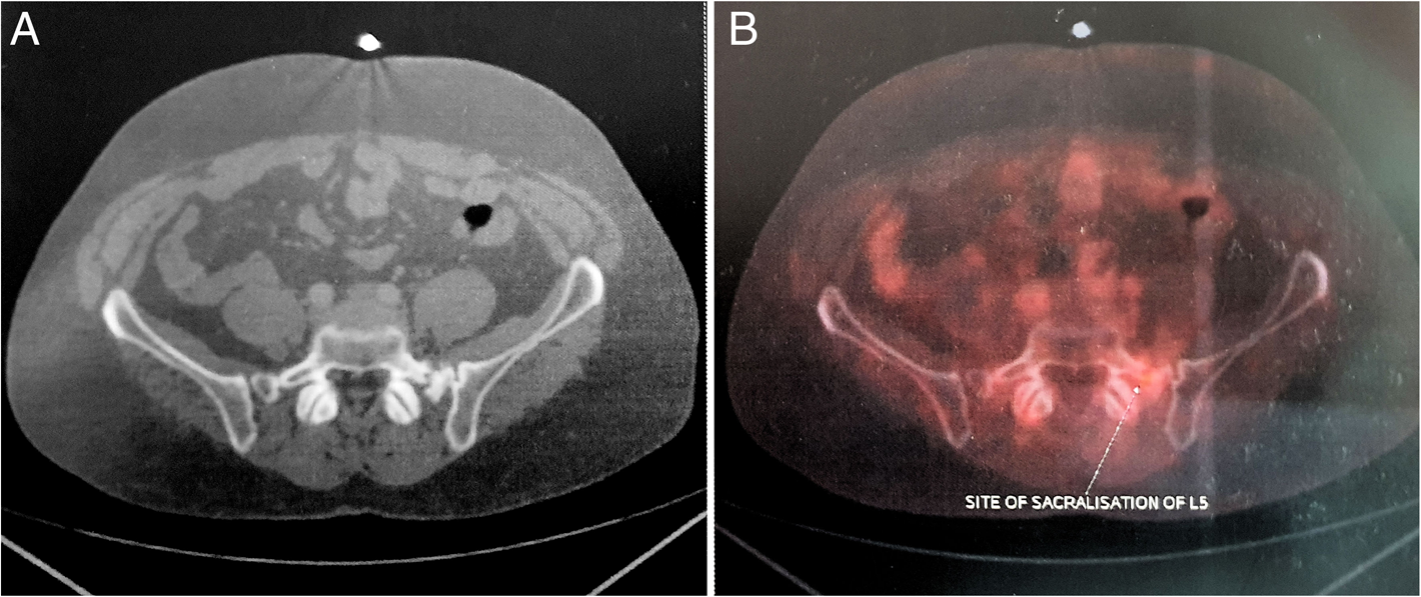
Follow-up FDG-PET in 2019. **a** Residual sclerosis in the left sacral ala **b** with the resolution of previous FDG uptake at L5-S1 facet

**Fig. 7:**
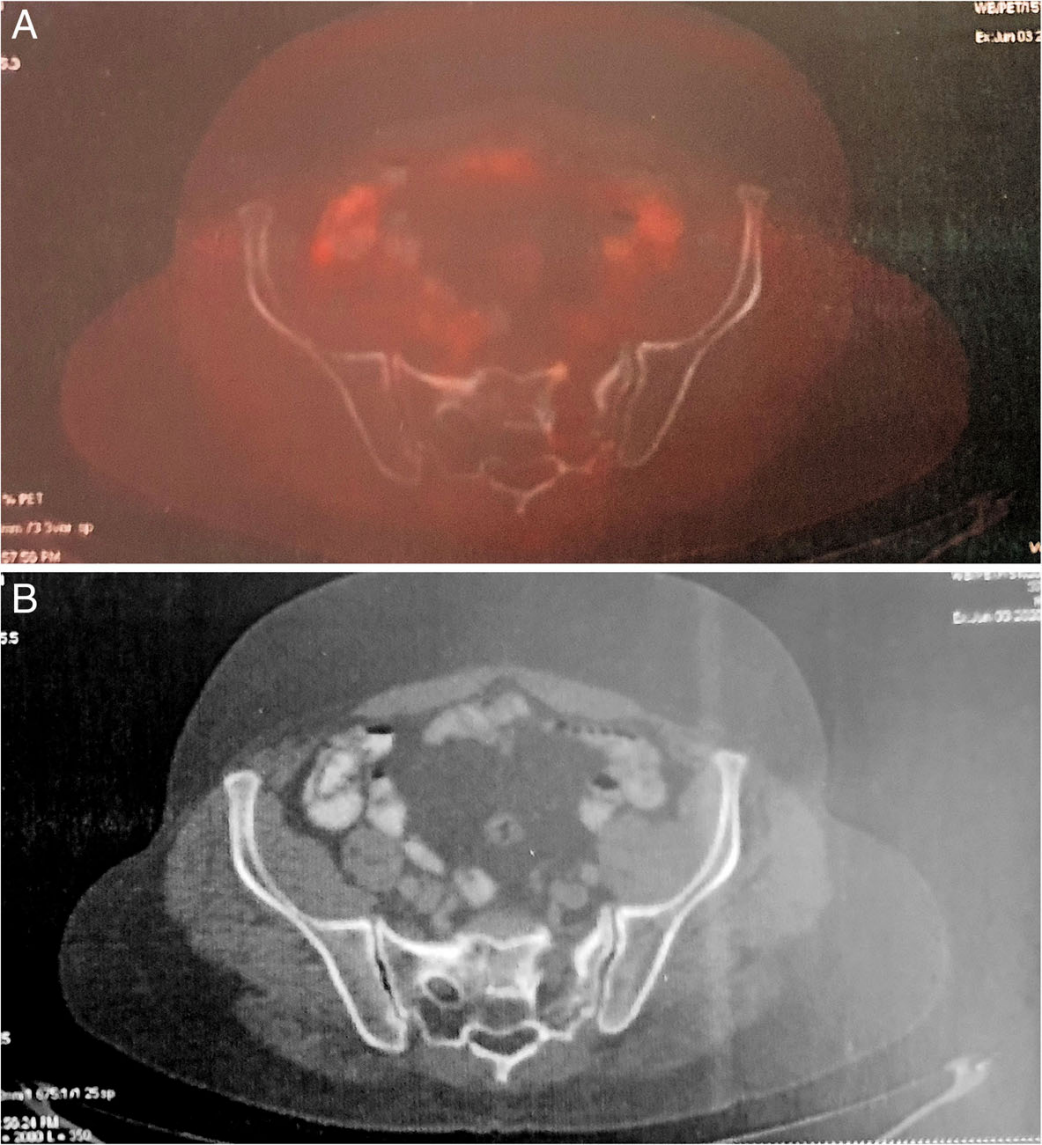
Follow-up FDG-PET in 2020. **a** Subtle sclerosis in the left sacral ala **b** with no FDG avid lesions

Post-treatment FDG-PET was repeated after 3 weeks, which showed metabolically inactive sclerosis at the left sacral ala scored as 1 on Deauville criteria using a 5-point scale as per Lugano classification (Fig. 4). The entire treatment was completed in 2017. The patient was placed on regular 3 monthly follow-up and had no fresh complaints. Follow-up FDG PET done in 2018 showed a stable ill-defined small sclerotic lesion in the left sacral ala without significant FDG uptake, representing metabolically inactive disease. It also showed FDG uptake (SUV 4) at the site of sacralization of L5 (Fig. 5), which was not appreciated in pervious scans representing arthropathy due to altered biomechanics. The patient was asymptomatic and clinically no abnormality was noted. Follow-up FDG-PET in 2019 showed non-FDG avid ill-defined sclerosis in the left sacral region and resolution of previously visualized mild FDG at L5-S1 facet (Fig. 6). No fresh complaints were noted. Recent follow-up FDG-PET in June 2020 showed no significant changes compared to previous FDG-PET (Fig. 7). The patient is asymptomatic without obvious clinical abnormalities and has metabolic complete response 42 months after completion of treatment.

## Methods

A systematic search was made on PubMed using the string (“sacrale”[All Fields] OR “sacralisation”[All Fields] OR “sacralised”[All Fields] OR “sacralization”[All Fields] OR “sacralized”[All Fields] OR “sacrals”[All Fields] OR “sacrum”[MeSH Terms] OR “sacrum”[All Fields] OR “sacral”[All Fields]) AND (“lymphoma”[MeSH Terms] OR “lymphoma”[All Fields] OR “lymphomas”[All Fields] OR “lymphoma s”[All Fields]). The title and abstracts of all the articles were reviewed and for articles wherein no information was provided in abstract and the article that were finally included in review full articles were extracted. Data on age, gender, histological type, treatment, follow-up, and response to treatment was extracted. Data was tabulated.

## Results

**Fig. 8:**
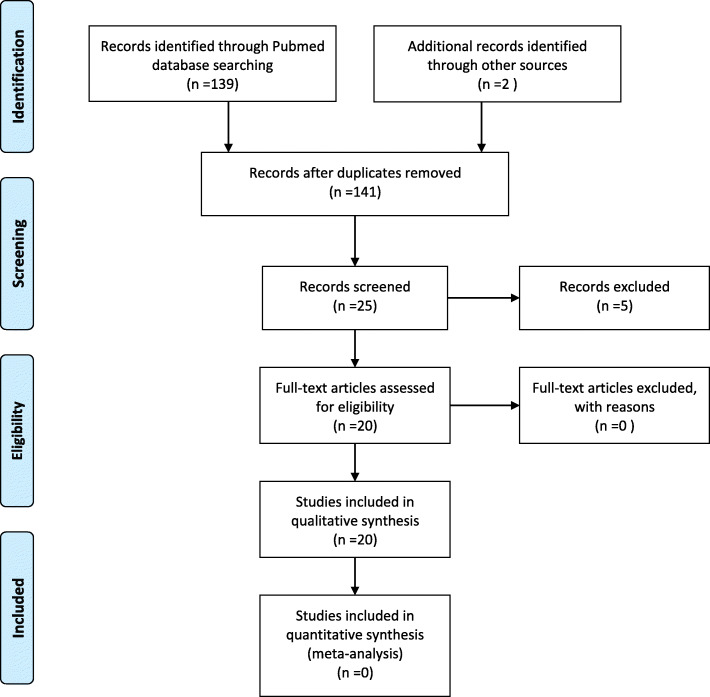
PRISMA flow chart of systematic search on sacral lymphoma

A total of 139 articles were extracted using the search string. Of these, 25 articles were selected and 116 articles were excluded as they did not report on primary sacral lymphoma (Fig. 8). After review of full text, another 5 articles were excluded as they did not report on the primary sacral lymphoma as per the definition reported in introduction. Of the 20 articles, one article reported 2 cases, and one reported 35 cases, while others reported one case each. In the case series of 35 cases, details of histology and treatment outcome were not reported. In total, 53 cases of sacral lymphoma were identified of which 7 were DLBCL, one DLBCL with EBV and myc translocation, one B cell NHL without further characterization, and 3 cases of Hodgkin’s disease while the majority were not characterized and were reported as lymphoma (NOS).

## Discussion

Primary bone lymphomas are rare constituting less than 2% of all lymphomas in the adult population. These tumors mostly involve the femur, other long bones of extremities, and pelvis [1, 2]. Primary tumors of the sacrum are rare, constituting 1–2% of all musculoskeletal tumors [15] and less than 7% of all primary spinal tumors [16]. Metastasis is the most common malignancy in the sacrum, and chordoma is the most common of all primary sacral tumors [17]. Primary bone lymphoma constitutes only 0.4% of all primary bone malignancy [18], of which primary lymphoma has been reported in 53 cases of diffuse large B cell lymphoma of sacrum which is an extremely rare presentation with only 11 reported cases in PubMed (Table 1). Usually, primary lymphoma of the bone occurs in the middle to elderly age group with male predominance [28]. Lymphoma (NOS) is the most common type encountered [29]. Common clinical symptoms are back pain with or without radiculopathy. Usual B type symptoms like fever, sweating, and weight loss are rarely encountered. The approximate size of the presentation is 2–7 cm, and laboratory findings are usually nonspecific. The most common finding on imaging is osteolytic bony destruction seen in 70% cases [30]. Differential diagnosis of imaging is metastasis, multiple myeloma, and other primary bone tumors. Usual MRI findings are “low signal intensity on T1 weighted images and high signal intensity on T2 weighted images, although not specific of lymphoma of bone” [10]. Sacral chordomas and chondrosarcomas on imaging show calcification specks. In the absence of osteolytic bone destruction and atypical MRI images, FDG-PET is a useful tool. Multiple myeloma and lymphoma have similar characteristics on MRI imaging, but multiple myeloma has no tracer uptake on isotope scan producing a cold spot. FDG-PET is also useful in disease staging and evaluating treatment response [31]. Histological and immunochemistry is used to confirm the diagnosis. On histology, bony lymphomas show atypical lymphoid cell proliferation. On immunohistochemistry, these tumors are positive for leukocyte common antigen (LCA) and CD20. Primary bone tumors from lymphoma can be differentiated on the basis of histology by their microscopic features for most neoplasms except for small cell osteosarcoma and Ewing’s sarcoma, which has to be excluded by IHC for CD99. Small cell carcinoma metastasis from the lung can also be excluded on IHC, as they are positive for cytokeratin and negative for leukocyte common antigen. Treatment of choice is R-CHOP (rituximab, cyclophosphamide, doxorubicin, vincristine, and prednisolone) chemotherapy regimen followed by radiotherapy. Patients with spinal cord or cauda equina compression features should undergo surgery for decompression and tissue diagnosis followed by radiotherapy [9]. In the present case report, the patient was referred to our department with complaints of low backache and radiculopathy which is quite a very rare presentation. Primary non-Hodgkin lymphoma localized to the bone has a better prognosis than in whom bone involvement is secondary to systemic disease [9]. Because of very few reported cases, and incomplete reporting and reporting after shorter follow-up, the incidence and prognosis of primary sacral lymphoma are not still very clear.

**Table 1:** Age, gender, pathological diagnosis, treatment, and outcome in patients with primary sacral lymphoma reported in literature till December 2020

Author and year [Ref]	Age	Gender	Histology	Treatment	Follow-up	Outcome
Nayil K et al. 2011 [9]	56	M	NHL-B cell no further categorization	S1-S2 laminectomy + RT	6 months	Well
Shimada A et al. 2013 [10]	85	M	Non GC type DLBCL, EBV, Myc translocation	R-CHOP	12 months	CR, disease free
Xu T et al. 2020 [13]	84	F	DLBCL	R-CHOP	5 months	CMR
Li GN et al. 2020 [6]	77	M	DLBCL	R-CHOP	1 month	On therapy
Ediriwickrima LS and Zaheer W 2011 [5]	67	M	DLBCL with lupus anticoagulant	R-CHOP	2 months	Disappearance of lupus anticoagulant
Wang J et al. 2020 [19]	35 cases Mean 46.2 years	15 M 20 F	Lymphoma (NOS)	Not described	Not described	NR
Liu JK et al. 2003 [7]	52	M	DLBCL	S2-S3 sacral laminectomy+ RCHOP + RT	4 months	Reduction in size, no metabolic imaging performed
Liu JK et al. 2003 [7]	64	M	DLBCL	3 cycles of CHOP followed by RT	13 months	Reduction in size, no metabolic imaging performed
Thornton E et al 2012 [20]	53	M	Lymphoma (NOS)	Not reported	Not reported	Not reported
Fourati N et al. 2017 [21]	24	–	HD	BEACOPP ×2 ABVD ×4 RT	15 months	CR
Ha-Ou-Nou F et al 2013 [22]	35	M	HD	ABVD	NR	NR
Ezenekwe AM et al 2004 [23]	50	M	Precursor B cell lymphoblastic lymphoma	Carmustine, cyclophosphamide, cytarabine, doxorubicin, etoposide followed by surgical excision	10	CR
Loh JK et al. 2005 [24]	6	M	Lymphoma (NOS)	Subtotal resection	11 years	CR
Hermann G et al. 1997 [25]	NR	NR	Lymphoma (NOS)	NR	NR	NR
Theodorou DJ et al 2000 [12]	58	M	Monostotic primary non-Hodgkin’s lymphoma of the bone with intrathecal involvement	Combination chemo Intrathecal MTX RT Peripheral blood stem cell transplant	1 year	CR
Chaari N et al. 2011 [4]	49	F	DLBCL	NR	NR	NR
Kirsch DG et al. 2005 [26]	14	F	HD	Two cycles of vincristine, prednisone, procarbazine, and doxorubicin followed by four cycles of cyclophosphamide, vincristine, prednisone, procarbazine + proton beam RT	2.5 years	CR
Ackerman L et al. 1994 [27]	36	M	Large cell lymphoma	NR	NR	NR
Tazi EM et al. 2009 [11]	45	M	DLBCL	CHOP +RT	10 years	CR, later developed histoplasmosis at same site
Amonkar AP et al. 2017 [3]	39	M	GC type DLBCL	RT followed by CHOP	NR	NR
Mally R et al. 2011 [8]	24	M	DLBCL	CHOP Intrathecal MTX RT	3 months	NR
Present case	36	M	DLBCL	RCHOP Maintenance R RT	36 months	CR

*CR* Complete response, *CMR* Complete metabolic response, *NR* Not reported, *HD* Hodgkin's disease, *GC* germinal center, *DLBCL* Diffuse large B cell lymphoma

## Conclusion

Primary sacral lymphoma is very rare entity and has imaging similarities with other tumors of sacrum. Definitive diagnosis is made only by HPE, and IHC is required for differentiating it from the rest of bony lesions. Hence, this case demonstrates the importance of insisting on core biopsy and not relying only on fine needle cytology if the diagnosis does not fit the overall clinical picture; non-Hodgkin's lymphoma has a good response to the R-CHOP regimen and radiotherapy while in a metastatic disease the outcome is dismal. Although few studies show better prognosis in primary bone NHL, the incidence and prognosis of primary sacral lymphoma are not still very clear because of very few reported cases.

## Learning points

- Primary sacral lymphoma is a rare differential diagnosis for primary sacral tumor.
- Definitive diagnosis is by biopsy and immunohistochemistry.
- Treatment options are R-CHOP regimen, radiation therapy, and surgical decompression.
- It is important to obtain a core biopsy and do immunohistochemistry in cases where the imaging and clinical picture does not support the provisional diagnosis.
- Depending upon cytology alone can be disastrous at times.

## Data Availability

Not applicable as all information and data are presented in the manuscript.

## References

[1] Ramadan KM, Shenkier T, Sehn LH, Gascoyne RD, Connors JM (2007). A clinicopathological retrospective study of 131 patients with primary bone lymphoma: a population-based study of successively treated cohorts from the British Columbia Cancer Agency. Ann Oncol..

[2] Salter M, Sollaccio RJ, Bernreuter WK, Weppelmann B (1989). Primary lymphoma of bone: the use of MRI in pretreatment evaluation. Am J Clin Oncol..

[3] Amonkar AP, Mallaiah B, Musthafa FB (2017). Primary lymphoma of the sacrum- a rare entity. Clin Oncol..

[4] Chaari N, Chebel S, Mahfoudh A, Drira A, Ali Jellali M, Moussa A (2011). Sacrum B cell-non-Hodgkin’s lymphoma complicating a chronic viral hepatitis C related to a blood exposure: a case report. Ann Biol Clin (Paris).

[5] Ediriwickrema LS, Zaheer W (2011). Diffuse large cell lymphoma presenting as a sacral mass and lupus anticoagulant. Yale J Biol Med..

[6] Li GN, Xiao L, Li L (2020). (18)F-FDG PET/CT imaging in a patient with solitary primary sacral lymphoma. Hell J Nucl Med..

[7] Liu JK, Kan P, Schmidt MH (2003). Diffuse large B-cell lymphoma presenting as a sacral tumor. Report of two cases. Neurosurg Focus..

[8] Mally R, Sharma M, Khan S, Velho V (2011). Primary lumbo-sacral spinal epidural non-Hodgkin’s lymphoma: a case report and review of literature. Asian Spine J..

[9] Nayil K, Makhdoomi R, Ramzan A, Malik R, Alam S, Wani A (2011). Primary sacral lymphoma: a case report and review of the literature. Turk Neurosurg..

[10] Shimada A, Sugimoto KJ, Wakabayashi M, Imai H, Sekiguchi Y, Nakamura N (2013). Primary sacral non-germinal center type diffuse large B-cell lymphoma with MYC translocation: a case report and a review of the literature. Int J Clin Exp Pathol..

[11] Tazi EM, Essadi I, Serraj K, Ichou M, Errihani H (2009). Sacrum histoplasmosis 10 years after NHL of the sacrum: a case report. Cancer Radiother..

[12] Theodorou DJ, Theodorou SJ, Sartoris DJ, Haghighi P, Resnick D (2000). Delayed diagnosis of primary non-Hodgkin’s lymphoma of the sacrum. Clin Imaging..

[13] Xu T, Fu W, Zhang X, Chen Y (2020). A case of primary sacral lymphoma evaluated by 18F-FDG PET/CT. Clin Nucl Med..

[14] Fletcher CDM, Unni KK, Mertens F. Pathology and genetics of tumours of soft tissue and bone in Pathology & genetics : tumours of soft tissue and bone edited by Christopher D.M. Fletcher, K. Krishnan Unni, Fredrik Mertens. Lyon: IARC Press; 2002. Available at https://www.ncbi.nlm.nih.gov/nlmcatalog/101184724.

[15] Capanna R (1997). Benign and malignant tumors of the sacrum. The Adult Spine: Principles and Practice.

[16] Llauger J, Palmer J, Amores S, Bagué S, Camins A (2000). Primary tumors of the sacrum: diagnostic imaging. AJR Am J Roentgenol..

[17] Epelbaum R, Haim N, Ben-Shahar M, Ben-Arie Y, Feinsod M, Cohen Y (1986). Non-Hodgkin’s lymphoma presenting with spinal epidural involvement. Cancer..

[18] Kelley SP, Ashford RU, Rao AS, Dickson RA (2007). Primary bone tumours of the spine: a 42-year survey from the Leeds Regional Bone Tumour Registry. Eur Spine J..

[19] Wang J, Li D, Yang R, Tang X, Yan T, Guo W (2020). Epidemiological characteristics of 1385 primary sacral tumors in one institution in China. World J Surg Oncol..

[20] Thornton E, Krajewski KM, O’Regan KN, Giardino AA, Jagannathan JP, Ramaiya N (2012). Imaging features of primary and secondary malignant tumours of the sacrum. Br J Radiol..

[21] Fourati N, Kanoun Belajouza S, Regaieg H, Khlif A, Bouaouina N (2017). Primary osseous Hodgkin’s lymphoma of the sacrum: a diagnostic and therapeutic challenge. Cancer Radiother..

[22] Ha-ou-nou FZ, Benjilali L, Essaadouni L (2013). Sacral pain as the initial symptom in primary Hodgkin’s lymphoma of bone. J Cancer Res Ther..

[23] Ezenekwe AM, Collins BT, Ponder TB (2004). Fine needle aspiration biopsy of precursor B-cell lymphoblastic lymphoma presenting as a sacral mass. A case report. Acta Cytol..

[24] Loh JK, Lin CK, Hwang YF, Hwang SL, Kwan AL, Howng SL (2005). Primary spinal tumors in children. J Clin Neurosci..

[25] Hermann G, Klein MJ, Abdelwahab IF, Kenan S (1997). MRI appearance of primary non-Hodgkin’s lymphoma of bone. Skeletal Radiol..

[26] Kirsch DG, Ebb DH, Hernandez AH, Tarbell NJ (2005). Proton radiotherapy for Hodgkin’s disease in the sacrum. Lancet Oncol..

[27] Ackerman L, Van Drunen M, Reyes CV (1994). Case report 836: malignant large cell lymphoma of sacrum. Skeletal Radiol..

[28] Rathmell AJ, Gospodarowicz MK, Sutcliffe SB, Clark RM (1992). Localized extradural lymphoma: survival, relapse pattern and functional outcome. The Princess Margaret Hospital Lymphoma Group. Radiother Oncol..

[29] Heyning FH, Hogendoorn PC, Kramer MH, Hermans J, Kluin-Nelemans JC, Noordijk EM (1999). Primary non-Hodgkin’s lymphoma of bone: a clinicopathological investigation of 60 cases. Leukemia..

[30] Dürr HR, Müller PE, Hiller E, Maier M, Baur A, Jansson V (2002). Malignant lymphoma of bone. Arch Orthop Trauma Surg..

[31] Yamamoto Y, Taoka T, Nakamine H (2009). Superior clinical impact of FDG-PET compared to MRI for the follow-up of a patient with sacral lymphoma. J Clin Exp Hematop..

